# Ultrastructural Analysis of Cancer Cells Treated with the Radiopharmaceutical Radium Dichloride ([^223^Ra]RaCl_2_): Understanding the Effect on Cell Structure

**DOI:** 10.3390/cells12030451

**Published:** 2023-01-31

**Authors:** Joel Félix Silva Diniz Filho, Aline Oliveira da Silva de Barros, Martha Sahylí Ortega Pijeira, Eduardo Ricci-Junior, Victor Midlej, Mariana Pelissari Monteiro Aguiar Baroni, Clenilton Costa dos Santos, Luciana Magalhães Rebelo Alencar, Ralph Santos-Oliveira

**Affiliations:** 1 Biophysics and Nanosystems Laboratory, Department of Physics, Federal University of Maranhão, São Luis 65065690, MA, Brazil; 2 Laboratory of Nanoradiopharmacy and Synthesis of New Radiopharmaceuticals, Brazilian Nuclear Energy Commission, Nuclear Engineering Institute, Rio de Janeiro 21941906, RJ, Brazil; 3 School of Pharmacy, Federal University of Rio de Janeiro, Rio de Janeiro 21941900, RJ, Brazil; 4 Laboratory of Structural Biology, Oswaldo Cruz Institute (FIOCRUZ), Rio de Janeiro 21040900, RJ, Brazil; 5 Department of Natural Science and Mathematics, Federal Institute of Education, Science and Technology (IFSP), Campus São Paulo, São Paulo 01109010, SP, Brazil; 6 Laboratory of Radiopharmacy and Nanoradiopharmaceuticals, State University of Rio de Janeiro, Rio de Janeiro 23070200, RJ, Brazil

**Keywords:** cell death, cell mechanism, targeted alpha therapy, radiopharmaceutical

## Abstract

The use of alpha-particle (α-particle) radionuclides, especially [^223^Ra]RaCl_2_ (radium dichloride), for targeted alpha therapy is steadily increasing. Despite the positive clinical outcomes of this therapy, very little data are available about the effect on the ultrastructure of cells. The purpose of this study was to evaluate the nanomechanical and ultrastructure effect of [^223^Ra] RaCl_2_ on cancer cells. To analyze the effect of [^223^Ra]RaCl_2_ on tumor cells, human breast cancer cells (lineage MDA-MB-231) were cultured and treated with the radiopharmaceutical at doses of 2 µCi and 0.9 µCi. The effect was evaluated using atomic force microscopy (AFM) and transmission electron microscopy (TEM) combined with Raman spectroscopy. The results showed massive destruction of the cell membrane but preservation of the nucleus membrane. No evidence of DNA alteration was observed. The data demonstrated the formation of lysosomes and phagosomes. These findings help elucidate the main mechanism involved in cell death during α-particle therapy.

## 1. Introduction

Alpha particles (α-particles) travel short distances (50–90 μm) due to their high energy [1]. In addition, α-particles are associated with high linear energy transfer (60–230 keV/μm), making these radionuclides excellent choices for therapeutic purposes [1]. The effects of alpha particles on cells can be explained by the interaction of ionizing radiation with biological matter. This radiation can have either a direct effect through the transfer of energy to biomolecules (DNA, lipids, proteins), leading to their ionization, or indirect effects, in which energy is transferred to water, which is then dissociated into reactive oxygen species (ROS), among which the hydroxyl radical HO° is the most reactive [2]. Alpha particles must cross the cell membrane and cytoplasm, including organelles and nuclear membrane, to reach the nucleus. A single α-particle passing into the cell nucleus has a 20–40% probability of killing the cell [3]. Recent studies have demonstrated some effects of ionizing radiation in these compartments and the contribution of these extranuclear effects to cell death [4,5,6,7].

Among the α-emitting radionuclides, radium-223 was the first radionuclide approved for clinical application in 2013 [8]. Radium-223 is used in the chemical form of [^223^Ra]RaCl_2_ salt. This radionuclide emits high-energy alpha particles with short-range (<100 μm), with low toxic effects on adjacent healthy tissue, and particularly bone marrow due to the short path of the alpha particles [9,10,11]. According to the Targeted Alpha Therapy Working Group theory [12], the high-energy emission of short-range alpha particles causes complex double-stranded DNA breaks.

[^223^Ra]RaCl_2_ is the first alpha-emitting radiopharmaceutical approved both in the United States and Europe (in 2013) for bone metastases from prostate cancer [13]. Alpha-emitting radiopharmaceuticals are highly toxic to tumor cells, with low toxicity to surrounding normal tissues [14]. From a safety point of view, this radiopharmaceutical is very safe, especially in terms of external exposure of staff and family members of patients treated with [^223^Ra]RaCl_2_ [15]. According to O’Dwyer and colleagues, alpha-emitting radiopharmaceuticals are highly cytotoxic anticancer drugs, allowing treatment of microscopic disease sites while sparing surrounding normal tissue [16].

Despite the widespread clinical use of [^223^Ra]RaCl_2_ (Xofigo) [8], there is little knowledge about the effect of α-particles on the ultrastructure of the cancer cells. In this study, we performed comprehensive analysis of breast cancer cells (MDA-MB-231) treated with [^223^Ra]RaCl_2_ using the most advanced microscopic techniques (atomic force microscopy and transmission electron microscopy), together with Raman spectroscopy analysis.

## 2. Materials and Methods

### 2.1. Breast Cell Culture

Human breast cancer cells (lineage MDA-MB-231) were obtained from the Rio de Janeiro Cell Bank (Rio de Janeiro, Brazil). The cells were routinely maintained in DMEM supplemented with 10% FBS, NaHCO_3_ (3.7 g/L), HEPES (5.2 g/L), L-glutamine (2 mM), fungizone (2.5 µg/mL), and penicillin and streptomycin (0.5 µg/mL each/together). Cells were incubated at 37 °C in a humidified atmosphere of 5% CO_2_ (CO_2_ incubator, series 3, Thermo Scientific, Waltham, MA, USA). The cells were grown to confluence in culture flasks.

### 2.2. Exposure to [^223^Ra]RaCl_2_ for Atomic Force Microscopy Analysis

MDA-MB-231 cells were cultured at a concentration of 5 × 10^6^ cells per well and seeded onto 13 mm circular glass coverslips in a 24-well plate. Then, the cells were treated in the presence or absence of a solution of [^223^Ra]RaCl_2_ (33.3 kBq; 0.9 µCi per well) for 24 h. After that, the cells were washed with PBS and fixed with a 3.7% formaldehyde solution for 10 min. The fixative solution was removed and cells were washed twice with ultrapure water and air-dried for morphology imaging and ultrastructure analysis. Two independent experiments were performed.

### 2.3. Atomic Force Microscopy

The atomic force microscopy analysis of cells was performed using an AFM Multimode 8 (Bruker, Santa Barbara, CA, USA) in PeakForce Quantitative Nanomechanics (QNM) mode in the air. Because we were handling radiopharmaceuticals, AFM imaging of living cells in a fluid medium was not feasible given the need to wait for the material’s decay, especially to observe the early effects of radiation on the cell membrane. As a result, fixed cells were analyzed to preserve this desired scenario. Probes (qp-HBC NanoSensors^TM^) with 0.5 N/m cantilever nominal spring constant and tip radius less than 10 nm were used in all measurements. All data were acquired with a 0.5 Hz scan rate and 0.5 kHz curve acquisition frequency. A total of 30 maps were analyzed for each group: (i) untreated MDA-MB-231 cells and (ii) cells treated with [^223^Ra]RaCl_2_ for 24 h. Each scan contained 65,536 force curves.

### 2.4. Roughness Analysis

The statistical roughness analysis was based on the height of each pixel in the image, analyzed from the height map. The parameter $R_{q}$s the mean square roughness, which is extremely sensitive to peaks and troughs and is defined by Equation (1) [17]:

(1) $$R_{q}=\sqrt{\frac{1}{N}\sum_{i=1}^{N}z_{i}^{2}},$$

where Z is each pixel height, and N is the total number of pixels (in our case, 65,536), with scan size standardized to 1 µm. This specific scan size (1 µm) was chosen to avoid height differences related to the entire cell topography. Therefore, the surface roughness obtained was associated with the ultrastructure’s of the cell membrane. Before roughness analysis, maps were pretreated with a third-order polynomial fit [18], minimizing extreme height differences and resulting in topography with more significant contributions from membrane structures.

### 2.5. Nanomechanical Analysis

The adhesion and stiffness of cells treated in the presence or absence of [^223^Ra]RaCl_2_ were calculated from all force curves. The adhesion force between the probe and the cell surface was obtained from retraction curves, determined at the minimum cantilever deflection value. This value represents the AFM probe’s resistance to leaving the sample surface. To calculate cell membrane stiffness, the slope values in the contact portion of the retraction curves were considered in intervals of 30–70% of deflection after the contact point of the tip with the sample. For both adhesion and stiffness data, the DMT model was employed in each force curve [19].

### 2.6. AFM Data Analysis

The normal distribution of AFM data according to a single criterion was assessed using ANOVA and Tukey’s post-test, with significance defined as *p* < 0.05. Statistical analyses and graphs of stiffness, adhesion, and roughness data were carried using the Origin software. Again, differences were considered significant when the *p*-value was <0.05.

### 2.7. Exposure to [^223^Ra]RaCl_2_ for Transmission Electron Microscopy Analysis

MDA-MB-231 cells were cultured at a concentration of 4 × 10^6^ cells per bottle. Then, the cells were treated with and without a solution of [^223^Ra]RaCl_2_ (74 kBq; 2 µCi) for 24 h. After that, cells were fixed in 2.5% glutaraldehyde in 0.1 M sodium cacodylate buffer (room temperature for 1 h) and post-fixed in 1% osmium tetroxide and 0.8% potassium ferrocyanide solution. The cells were dehydrated using an increasing concentration series of acetone (70, 90, and 100%), embedded in Epon resin, and polymerized at 60 °C. Ultrathin sections (60–70 nm thick) were obtained without staining. The morphological and ultrastructural analysis was performed using a Hitachi HT 7800 (Chiyoda City, Japan) transmission electron microscope. Two independent experiments were performed.

### 2.8. Raman Analysis and Spectral Processing

Twenty cell spectra from each group were measured over 600 cm^−1^ to 1800 cm^−1^ from the central region using a Horiba Jobin-Yvon spectrometer (model T64000) (Horiba, Kioto, Japan) with a CCD (charge coupled device) detection system, cooled with liquid nitrogen, using a laser at 532 nm. All measurements were obtained with single-mode backscattering geometry. Visualization of the sample’s surface was performed using an Olympus microscope (Shinjuku, Tokyo, Japan) with a video camera attached. To focus the beam on the surface, we used a 100× objective lens. Data processing was performed using the LabSpec6 software version 6.4. The narrow spikes caused by cosmic rays were removed in sequence, and the varying fluorescence background and glass substrate were estimated using fifth-order polynomial fitting and subtracted. Each spectrum was smoothed using a polynomial smoothing algorithm before the analysis.

### 2.9. Spectral Statistical Analysis

Principal component analysis (PCA) was used on the spectra dataset. It is a statistical method that can reduce the dimension of the data while accounting for most of the variance in the original data. The spectra were analyzed according to the method of Y. H. Ong et al. [20], in which analysis of variance is applied to the scores of the first ten principal components to determine which PC has significant differences in the mean scores between the two groups of cells, using the OriginLab software version 9.95.

## 3. Results

**Figure 1:**
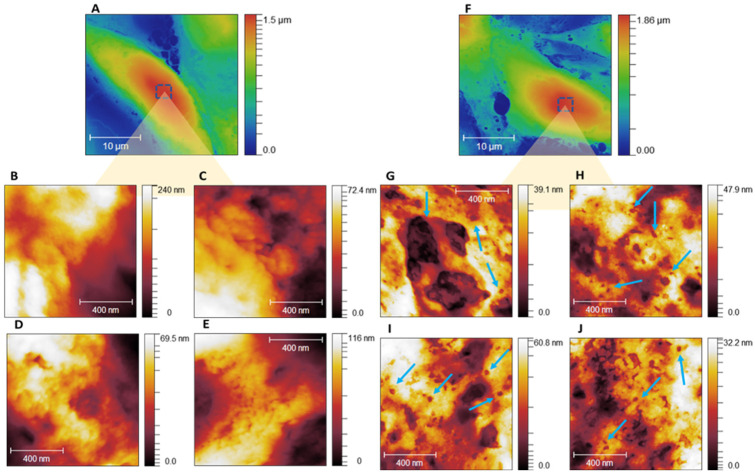
AFM topographic maps of the ultrastructure of MDA-MB-231 cells treated and not treated with [^223^Ra]RaCl_2_. (**A**): 30 × 30-micrometer map of cells untreated and (**F**) treated with [^223^Ra]RaCl_2_. The squares represent the regions where the zoom was performed to analyze the membrane’s ultrastructure: the cell’s highest areas (cell body). (**B**–**E**,**G**–**J**) show, respectively, a 1 × 1 µm zoom of the membrane of untreated and treated cells. The arrows in (**G**–**J**) indicate the nanoholes formed by the collision of alpha particles with the cell membrane. (**A**) shows the bar graph of the R_q_ values of the untreated sample (control) and [^223^Ra]RaCl_2_-treated sample. The result reveals a reduction of R_q_ values of the treated sample (11.73 ± 1.25 nm) in comparison with the control group (20.78 ± 3.35 nm), suggesting that the treatment with [^223^Ra]RaCl_2_ promotes wear of the membrane surface. (**B**) compares the valley counts of the samples (111.8 ± 22.88 and 192.73 ± 23.25, control and treated groups, respectively). The greater number of depressions observed in the treated samples further indicates the damage caused by the interaction of alpha particles with the cell membrane.

High-resolution AFM images of MDA-MB-231 cells revealed ultrastructural changes in the cell membrane. To ensure that the differences observed were indeed related to the effects of the [^223^Ra]RaCl_2_ treatment, numerous scans were performed (60 total), revealing changes in the membrane of the treated cells, as exemplified in Figure 1. By comparing 1 micrometer scans of the membrane of untreated cells (Figure 1C) with the membrane of cells treated with [^223^Ra]RaCl_2_ (Figure 1D), it was possible to observe the removal of membrane components causing defects in the membrane structure (regions indicated by the blue arrows). This result suggests that the interaction of alpha radiation with the cell membrane, in addition to promoting the known damage to the DNA chain, also causes wear to the external structure of the cell membrane.

**Figure 2:**
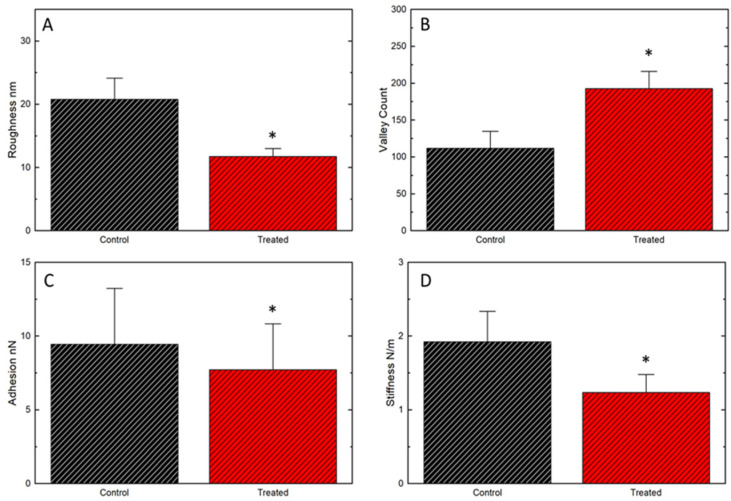
Quantitative data on ultrastructural and nanomechanical properties of the cell membrane. (**A**): mean square roughness between untreated and treated samples (20.78 ± 3.35 nm and 11.73 ± 1.25 nm respectively); (**B**): valley count (111.8 ± 22.88 and 192.73 ± 23.25, control and treated group respectively); (**C**): Membrane surface adhesion (9.45 ± 0.1 nN and 7.71 ± 0.08 nN (SD)); (**D**): Membrane stiffness (1.92 ± 0.4 N/m and 0.77 ± 0.6 N/m (SD)). * *p* < 0.05 in comparison with the control group.

Interestingly, for all sets of images analyzed, a decrease in the height differences was observed according to the scale bars of the treated samples compared to the untreated ones in the zooms of the membrane images. This result motivated us to investigate quantitative changes in membrane ultrastructure. Taking advantage of AFM’s ability to produce three-dimensional data from the analyzed topography, we analyzed the samples’ mean square roughness and valley counts, as shown in Figure 2.

Moreover, adhesion forces between the AFM probe and the cell membrane (Figure 2C) showed significantly lower values in the treated (7.71 ± 0.08 nN) group in comparison with the untreated group (9.45 ± 0.1nN). These variations in probe-sample interaction represent changes in the cell surface between treated and untreated groups, associated with the incidence of the alpha radiation from radium-223.

The surface stiffness of the cell membrane was also evaluated after treatment, showing reduction (0.77 ± 0.6 N/m) compared to the control group (1.92 ± 0.4 N/m). The decrease in the local stiffness of the membrane reinforces the hypothesis of alteration of the membrane components. Changes in the distribution and density of membrane components result in a difference in the local stiffness of the cell membrane [20,21].

All quantitative AFM analyses showed significant statistical differences based on extremely robust statistics: 30 maps were analyzed for each group and 65,536 force curves were obtained for each map. The adhesion force and local stiffness were calculated for each curve. Thus, it is evident that the differences observed in the ultrastructure and cellular nanomechanics were related to the treatment.

**Figure 3:**
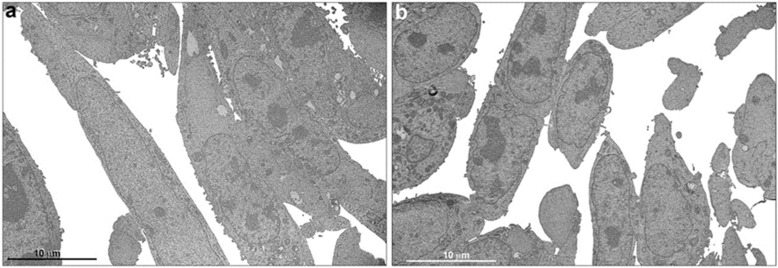
Transmission electron microscopy, general view of breast tumor-derived cells. Ultrastructural aspects of unexposed MDA-MB-231 cells (**a**) and cells exposed to [^223^Ra]RaCl_2_ (**b**). Note that in both situations the content of the nuclei is similar. There is no ultrastructural evidence of increased heterochromatin after radiation.

**Figure 4:**
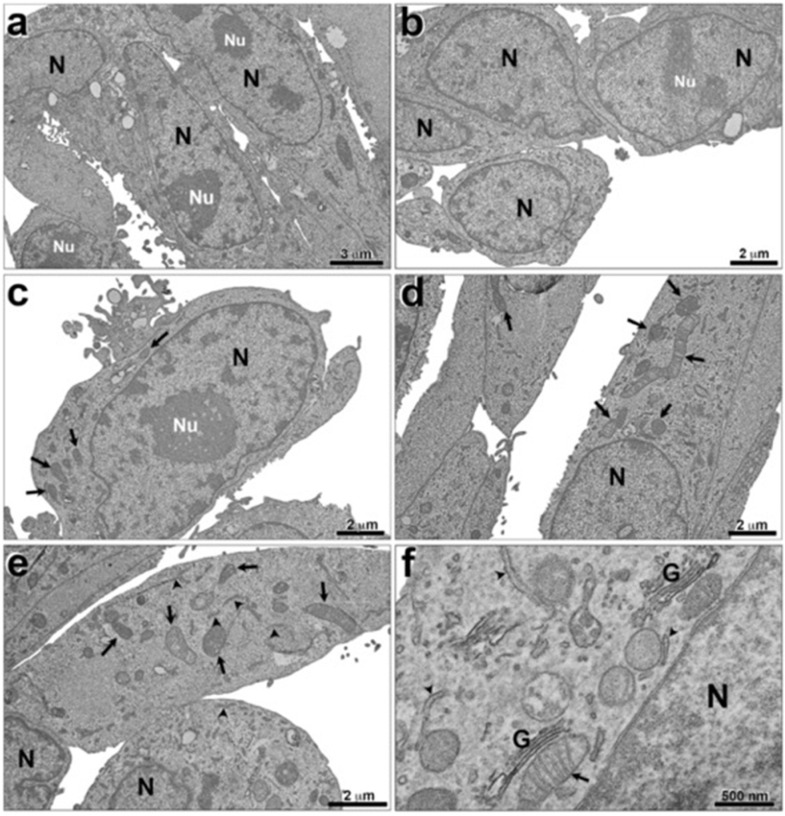
Ultrastructural analysis of breast tumor cells not exposed to [^223^Ra]RaCl_2_. It is possible to observe a preserved cytoplasm (**a**,**b**), mitochondria (arrows) (**c**–**f**), endoplasmic reticulum (arrowhead), and a Golgi complex (**f**). N, nucleus; Nu, nucleoli; G, Golgi complex.

A general analysis of the ultrastructural aspects of breast cancer cells revealed no difference in nuclear content when exposed and not exposed to alpha radiation (74 kBq; 2 µCi) for 24 h (Figure 3). The transmission electron micrographs of MDA-MB-231 cells not exposed to [^223^Ra]RaCl_2_ presented very close connections between their plasma membranes (Figure 4a–c). Nonetheless, unexposed cells had preserved nuclei with well-developed nucleoli (Figure 4a,c). It is also possible to note a well-conserved and homogeneous cytoplasm and its organelles (mitochondria, endoplasmic reticulum, and Golgi complex) (Figure 4a–f). The endoplasmic reticulum is dispersed through the cytoplasm with no accumulations. The mitochondria are located randomly in the cytoplasm and well-preserved cristae (Figure 4f).

**Figure 5:**
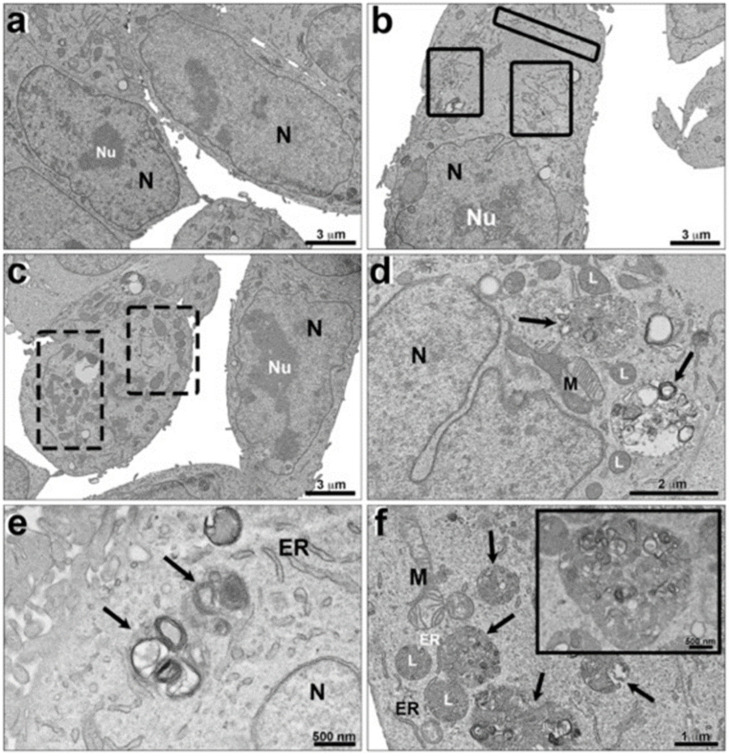
Ultrastructural analysis of breast tumor cells exposed to [^223^Ra]RaCl_2_. It is possible to observe very regular and preserved nucleus (**a**–**d**); higher amount of endoplasmic reticulum (rectangles with solid lines) (**b**) and of mitochondria (rectangles with dashed lines) (**c**). Note the presence of membrane surrounding structures, which compound the autophagosome (arrows) (**d**–**f**). A detailed phagosome is showed in (**f**). N, nucleus; Nu, nucleoli; M, mitochondria; ER, endoplasmic reticulum; L, lysosomes.

The cells exposed to [^223^Ra]RaCl_2_ (74 kBq; 2 µCi) for 24 h contained very regular and well-preserved nuclei as well as nucleoli (Figure 5a–d), very similar to the group of cells not exposed (Figure 5a–c). Moreover, the nuclear envelope was intact, displaying double-bonded membranes (Figure 5d,e). The chromatin was regular, and no heterochromatin was observed. Most DNA was in euchromatin form (Figure 5a–d).

**Figure 6:**
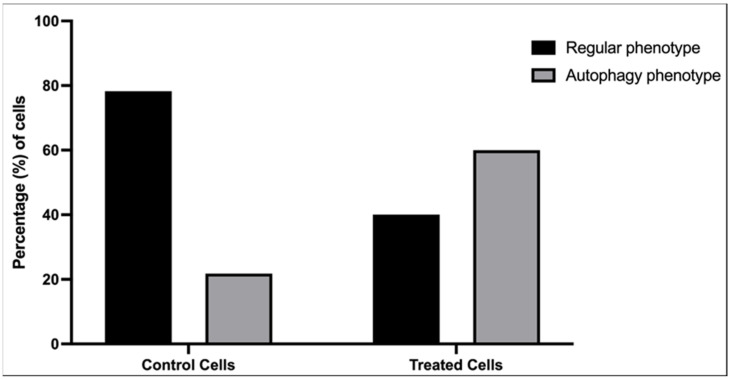
Quantitative analysis of autophagy phenotype in breast cancer cells after [^223^Ra]RaCl_2_ exposure. Thirty random fields were counted using transmission electron microscopy of exposed and unexposed cells. There was an increase of autophagic features in treated cells.

The images showed enlarged endoplasmic reticulum (Figure 5b) and mitochondria (Figure 5c). The results showed that the exposure to alpha radiation promoted the classic pathway of autophagy (Figure 5d–f) with the formation of autophagosomes and lysosomes. In Figure 5f, it is possible to observe these autophagosomes with autophagic signs of death. Quantitative analysis was performed to evaluate the expression of autophagic phenotype between the cells that were exposed and not exposed to radiation (Figure 6). Thirty random fields were observed by transmission electron microscopy and the chosen magnification for analysis was standardized to the limit of resolution, where the autophagy ultrastructural features could be observed. Figure 6 shows an increase of autophagy phenotype in MDA-MB-231 cells exposed to radiation in comparison with unexposed cells (Figure 6).

The Raman spectroscopy technique was used in this study to investigate the vibrational alterations of molecular groups promoted by the radiopharmaceutical in the cells.

**Figure 7:**
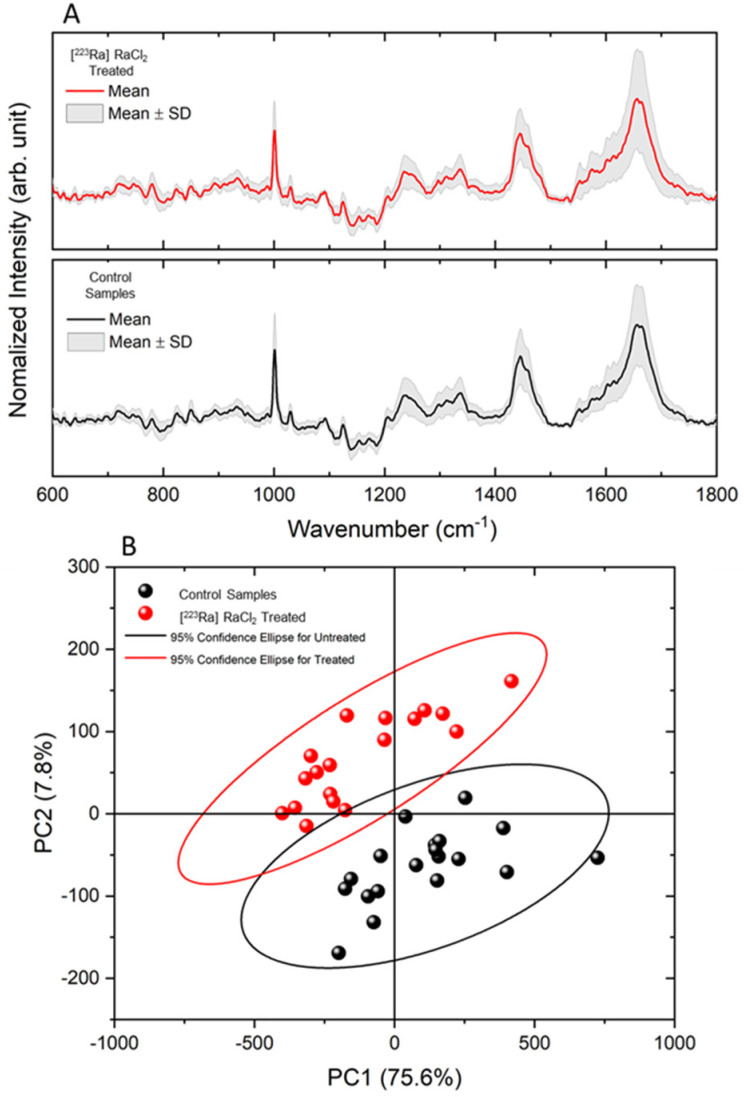
Raman spectroscopy. (**A**) Mean spectrum of MDA-MB-321 cells treated and untreated with [^223^Ra]RaCl_2_. (**B**) PCA showing the differentiation between groups with a total variance of 83.4%.

Figure 7 shows the results of Raman spectroscopy and PCA comparing treated and untreated cells. Figure 7A displays the average spectra of each group and the data dispersion for the 20 spectra obtained for each group. No significant differences were observed between the mean spectra, and there was no appearance or deletion of vibrational modes. Figure 7B exhibits the PCA results of these spectra. Although the average spectra did not show significant changes, the PCA demonstrated that 83.4% of the total variance was reflected in the first two principal components, indicating the differences between the groups. The main modes that contribute to this differentiation are, in order of relevance, around 1003 cm^−1^, 1446 cm^−1,^ and 1659 cm^−1^.

## 4. Discussion

The results indicated massive damage to membrane cells. The destruction of these cells can have important consequences on cell survival. According to Dias and Nylandsted [22], the maintenance of plasma membrane integrity is essential for cell viability and functioning. In addition to acting as a physical barrier between extracellular and intracellular matrices, the plasma membrane is highly dynamic, by connecting intracellular signaling cascades and extracellular signals, allowing cell communication with the surrounding environment. Plasma membrane damage threatens cell survival. [22,23]. Bertolet et al. [24] reported that the effect of ionizing radiation is not well known and that *α*-particles can affect biological organisms directly or indirectly.

Although the literature states that *α*-particles are responsible for DNA damage (irreparable DNA double-strand breaks) and, consequently, cell death [25,26], our data indicated no effect on DNA after the 24-h treatment with 74 kBq-[^223^Ra]RaCl_2_. This fact is corroborated by the demonstrated integrity of the nuclear membrane. According to Kidiyoor et al. [27], the nuclear envelope (membrane) is responsible for mechanically protecting the genome integrity, nuclear architecture, chromatin dynamics, gene expression, and cell migration and differentiation. Ruptures in the nuclear envelope (NE) in non-transformed cells are often associated with senescence and aging, reducing cell proliferation. However, in tumor cells, the damage to the nuclear membrane increases the potential for invasion and metastasis [28,29,30]. Recently, Nader GPF et al. (2021) demonstrated that nuclear membrane rupture induces an invasive phenotype in human breast carcinoma [31]. The maintenance of the integrity of the nuclear membrane is an indication of DNA integrity. Another sign that the DNA was preserved after the treatment with [^223^Ra]RaCl_2_ was the observation of the presence of chromatin and no formation of heterochromatin. Although Svetličič et al. [32], studying fractioned radiation exposure of breast cancer cells to alpha and gamma radiation, demonstrated the formation of heterochromatin and stemness properties induced by fractionated radiation exposure (alpha and gamma), our data do not confirm this finding, since we observed that alpha radiation did not influence the chromatin dynamics. Dense chromatin states, known as heterochromatin, have been easily observed by transmission electron microscopy (TEM) [33,34]. Severe chromatin damage usually forms large aggregates of DNA that are seen as heterochromatin by electron microscopy [35]. We observed no difference between the nuclear content of exposed and unexposed cells, revealing that [^223^Ra]RaCl_2_ exposure does not enhance heterochromatin of DNA. While some studies have detected DNA degradation using microscopic methods [35,36], this degradation has been correlated with biochemical data. Therefore, our data suggest there is no increase in heterochromatin. Still, due to the lack of comparison with biochemical techniques, it is impossible to state that this did not occur in short stretches of DNA.

Although our results demonstrated no significant effect on DNA, a better technique, such as gamma 2HAX, should be used to better evaluate the effect on DNA. In addition, it is important to notice that according to Van der Doelen [37], [^223^Ra]RaCl_2_ produces irreparable DNA damage, which, subsequently, can result in cell death through apoptosis. Marques et al. [38] corroborated this information.

The Raman spectroscopy analysis strongly corroborated these findings. When a molecular group changes, the vibrational modes relative to it are also altered. This fact can be verified by changes in the Raman spectrum’s peak intensity, position, and broadening. In this manner, one can determine which molecular groups of the structure are more sensitive to the effects of the radiopharmaceutical agent, providing a better understanding of the cell apoptosis process. We observed that the drug acted subtly on the molecular structure of the cells, so it was challenging to visually identify changes in the spectra of untreated and treated samples. However, with the aid of multivariate analysis using PCA, it was possible to determine the alterations in the vibrational modes, enabling a clear distinction between treated and untreated groups. According to the principal component analysis (PCA) of the spectra, it was possible to observe that treated and untreated groups were distinct, proving that the interaction of [^223^Ra]RaCl_2_ with the cells modified them, distinguishing them from the cells of the control group. The main differences that contributed to explain the 83.4% variance in PCA was associated with modes around 1003 cm^−1^, 1446 cm^−1,^ and 1659 cm^−1^. According to J. Xu et al. [39], these modes are associated with lipids and proteins (1446 cm^−1^ and 1659 cm^−1^) and phenylalanine (1003 cm^−1^). This result agrees with the AFM data, since the changes observed on the surface of the membrane would lead to changes in the bands of proteins and lipids, the main components of the cell membrane. As observed in the AFM images, membrane changes caused by alpha radiation was associated with changes in Amide I and lipid bands.

Finally, the results showed the formation of lysosomes and phagosomes, representing one of the main mechanisms involved in cell death [40]. According to Noguchi et al. [40], the Akt-mammalian target of the rapamycin complex (mTORC1) pathway regulates the proton pump [vacuolar H^(+)^-adenosine triphosphatase ATPase (v-ATPase)] in the lysosomal membrane, which transports protons from the cytosol, thereby regulating the lysosomal acidification process required to induce autophagy. We believe that alpha-particles, composed of two protons, promoted proton pump imbalance, leading to substantial lysosomal formation and autophagy.

## 5. Conclusions

The data obtained in this study showed that cancer cells, when treated with alpha particles, suffer intense degradation of the cell membrane with preservation of the nucleus envelope at the dose evaluated after 24 h of treatment. In addition, no formation of large extensions of heterochromatin was observed. Our data improve our understanding of the effects of alpha particles, especially the radiopharmaceutical [^223^Ra]RaCl_2_, at the cellular level.

## Data Availability

Not applicable.
